# The Use of POCUS-Obtained Optic Nerve Sheath Diameter in Intracerebral Hemorrhage

**DOI:** 10.24908/pocus.v8i2.16563

**Published:** 2023-11-27

**Authors:** Alireza Nathani, Shekhar A Ghamande, Sarita Kambhampati, Braden Anderson, Matthew Lohse, Heath D White

**Affiliations:** 1 Baylor Scott & White Health, Baylor College of Medicine Temple, TX USA

**Keywords:** Intracranial Hemorrhage, Optic nerve sheath diameter, ocular ultrasound

## Abstract

**Background:** Intracerebral hemorrhage (ICH) is associated with high morbidity and mortality. ICH causes increased intracranial pressure (ICP), leading to brain herniation as the disease progresses. Neurological physical exam and monitoring of the disease progression can be challenging due to the impaired consciousness and routine clinical management in this patient population. Given the continuity of the intracranial cavity with the optic nerve subarachnoid space, an increased ICH leads to distension of the optic nerve sheath. We herein examined the correlation between the ICH volume and the optic nerve sheath diameter (ONSD) measured by point of care ultrasound (POCUS). **Methods:** Patients with ICH diagnosed with a head computed tomography (CT) scan were prospectively enrolled in this study. A portable ultrasound was used to measure the (ONSD); the volume of ICH hematoma, the Acute Physiology And Chronic Health Evaluation IV score, and the Intracerebral Hemorrhage score were collected. A Spearman rank correlation coefficient test was used to assess the relationship between continuous variables. A Wilcoxon rank sum test was used to assess differences in continuous variables between two groups. A p-value less than 0.05 was deemed as statistically significant. **Results:** A total of 28 subjects were enrolled. A moderate positive correlation was detected between hemorrhage volume and the average ONSD (correlation = 0.4214, p = 0.0255). A weak positive correlation was detected between average ONSD and APACHE IV (correlation = 0.2347, p = 0.2294). A weak moderate positive correlation was detected between average ONSD and ICH score (correlation = 0.1160, p = 0.5566). **Conclusions**: In this study we demonstrate that ONSD is moderately correlated with hematoma size. A potential application may include serial measurements of the ONSD with ultrasound. This may offer a quick, non-invasive technique that can be used in an intracerebral hemorrhage to monitor the stability or expansion of a hematoma indirectly, and potentially catch a catastrophic event like cerebral herniation.

## Introduction

Intracerebral hemorrhage (ICH) results in spontaneous bleeding into the brain. In the United States, it accounts for 10-15% of all strokes 1. It is associated with very high morbidity and in-hospital mortality of 32.4% 2. In adults, the intracranial compartment is protected by the skull. The contents of the intracranial compartment include brain parenchyma, cerebrospinal fluid, and blood. Homeostasis of all three components is required to keep intracranial pressure less than 15mmHg. In an ICH, additional blood is now enclosed in the cranium which can apply pressure to normal structures of the brain. Additionally, cerebral edema will occur through a variety of mechanisms including cytotoxic cell injury that will cause further compression on normal structures. The neurological physical exam is crucial in detecting cerebral edema, particularly compression of the brain stem, which regulates most of the body's automatic functions that are essential for life. Hence, the rapid diagnosis of increased intracranial pressure is crucial for diagnostic and therapeutic purposes in this population. The optic nerve, also known as cranial nerve II, transmits visual information from the retina to the brain. The optic nerve is surrounded by the optic nerve sheath, which is directly linked to the intracranial subarachnoid space. An increase in intracranial pressure (ICP) causes cerebral spinal fluid to move from the intracranial cavity into the optic subarachnoid space, thereby resulting in distension of the optic nerve sheath and widening of its diameter 3. Given this physiologic relationship, our team was interested in determining if there was a direct correlation between the volume of hematoma in an ICH and the size of optic nerve sheath diameter (ONSD) as measured by point of care ultrasound (POCUS). For reference a normal ultrasound obtained ONSD in males is 4.66mm and 4.47mm in females 4, although there are numerous studies that have reported different values of a normal ONSD.

## Materials and Methods

This study was a prospective trial conducted at Baylor Scott & White Medical Center – Temple, TX. The study protocol was approved by and conducted under the supervision of the Baylor Scott & White Research Institute Institutional Review Boards. The study was funded by the department of neurology. Twenty-eight patients were enrolled in this study. All patients included were over the age of 18 and had the diagnosis of ICH confirmed with a non-contrast head computed tomography (CT) scan. All patients were admitted to the neurointensive care unit and managed according to the standard of care. Within six hours of the patient’s admission, informed consent would be obtained from the patient or legally authorized representative to obtain POCUS images of their optic nerves. Excluded were patients with obvious ocular pathology including ocular trauma, foreign bodies in the eye, or a glass eye. Also excluded were subarachnoid hemorrhages and ischemic strokes. 

**Figure 1:**
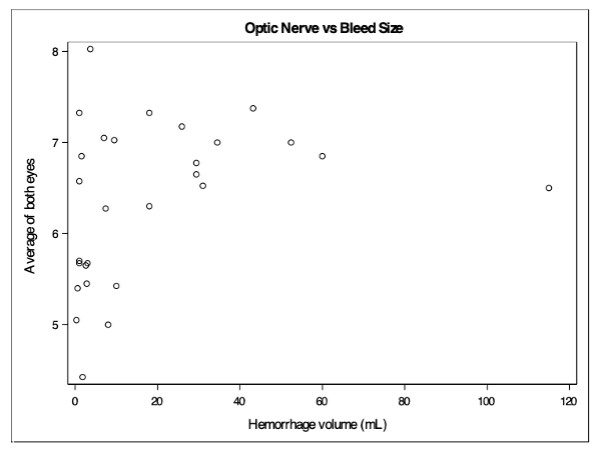
Posterior eye showing optic nerve sheath and ONSD.

**Figure 2:**
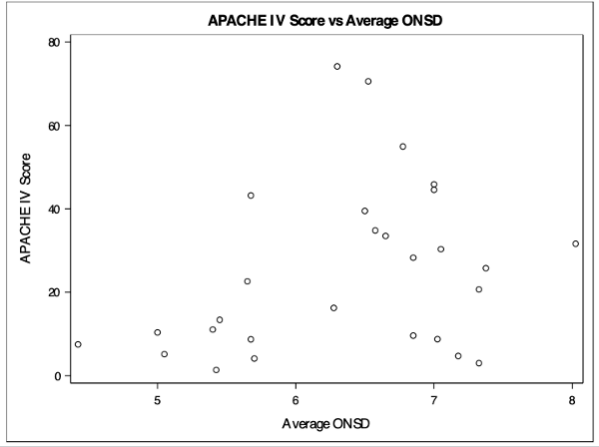
Ultrasound image showing components of optic nerve sheath exam.

A portable ultrasound device (Sonotsite) was used to measure the optic nerve sheath diameter. A high frequency (5-14 MHz) probe was used to obtain images. A thin film of gel was applied to each eyelid. The optic nerve was visualized in transverse and longitudinal planes. We measured from the inner edge to inner edge of the optic nerve sheath 3mm behind the optic globe 3, 5. Figure 1 is a pictorial view describing the relevant anatomic components of the posterior eye and Figure 2 illustrates what the operator sees while performing an exam 5, 6. After the exam was complete, the excess gel was wiped off the patient. Transverse and longitudinal values were obtained for each optic nerve. These values were averaged, and a single value was obtained for the left and right optic nerve. Other data collected include the volume of ICH (calculated based on the head CT scan), the APACHE (Acute Physiology And Chronic Health Evaluation) IV score, and the ICH (intracerebral hemorrhage) score. A Spearman rank correlation coefficient test was used to assess the relationship between continuous variables. A Wilcoxon rank sum test was used to assess differences in continuous variables between two groups. Statistical significance was set at p<0.05. All statistical analyses were performed in SAS 9.4.

## Results

**Figure 3:**
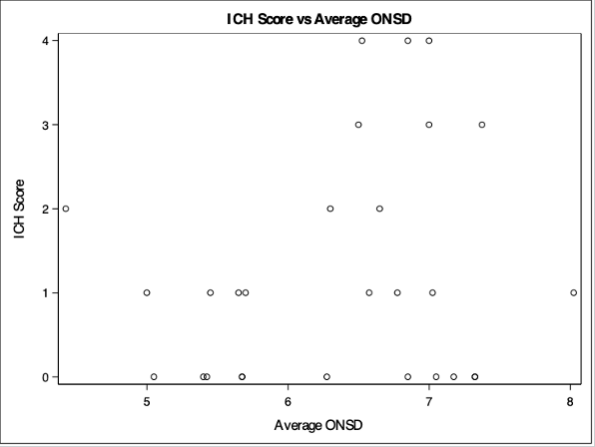
Average ONSD vs hemorrhage volume.

**Figure 4:**
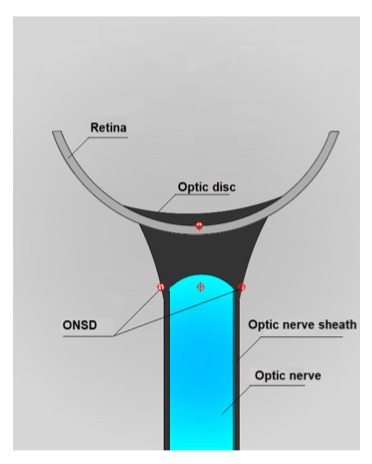
APACHE IV vs average ONSD.

**Figure 5:**
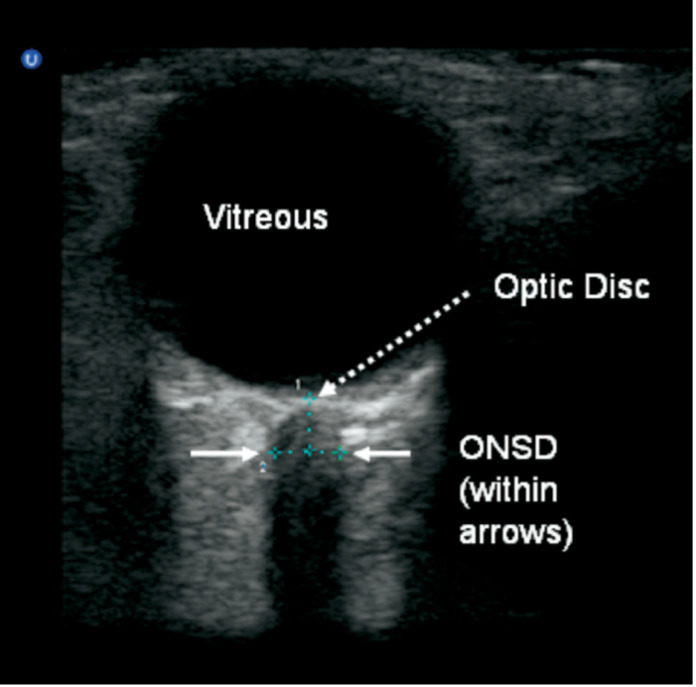
ICH score vs average ONSD.

A total number of 28 subjects were enrolled in the study. There were 16 females and 12 males with a mean age of 67.5 years (mean: 67.5, SD: 15.0). The mean optic nerve sheath diameter was 6.36mm. The median hemorrhage volume was 7.70mL. A moderate positive correlation was detected between hemorrhage volume and the average ONSD (correlation = 0.4214, p = 0.0255, Figure 3). A weak positive correlation was detected between average ONSD and APACHE IV (correlation = 0.2347, p = 0.2294, Figure 4). A weak moderate positive correlation was detected between average ONSD and ICH score (correlation = 0.1160, p = 0.5566, Figure 5). 

## Discussion

Growth of an intracranial hematoma in the first twenty-four hours is an independent predictor of mortality and poor outcome 4. The neurological exam is crucial in determining the progression of swelling in the brain. However, the accuracy and reliability of neurological physical exams are frequently limited in these particular type of patients. Neuroanatomical areas maintaining alertness or consciousness are oftentimes damaged, and patients undergo endotracheal intubation to protect the airway and routinely require analgesia to succumb agitation. All of these can confound the neurological exam. Additionally, there are a number of teams working with the patient including the critical care team, stroke neurology, and neurosurgery, each of which may record different physical exam findings. Using POCUS to measure the ONSD in these specific situations can be an objective adjunct to the physical examination. A common scenario where we foresee this being useful is during the first twenty-four hours of admission for ICH when hematoma expansion risk is greatest. Performing serial optic nerve exams with ultrasound may be useful to observe a trend in the ONSD. A significant increase in the ONSD compared to previous values can alert the physician that the ICH may be expanding, and that further imaging may be required to assess hematoma stability. Moreover, in patients who are critically ill and unstable for transport, bedside ONSD examination can potentially be used by clinicians to assess for hematoma expansion. Finally, invasive intracranial pressure monitoring devices may be contraindicated for various reasons including but not limited to coagulopathy. A bedside exam of the optic nerve in this situation may be helpful for monitoring. A weak positive correlation was also detected between average ONSD and ICH score as well as APACHE IV score. This weak correlation is likely because several components of the ICH score may not corollate with hematoma size, such as presence of intraventricular hemorrhage, age greater than 80, and infratentorial location of hemorrhage. The APACHE IV score uses variables derived from values from the first twenty-four hours of ICU admission. It is a hospital mortality predictor. There was a weak positive correlation that was not statistically significant. The variables included in the APACHE IV also contain chronic health conditions and lab chemistries, which may or may not be directly related to the ICH. The ICH score is based on age and CT findings and has been validated to estimate mortality. We found a weak moderate positive correlation between ICH score and average ONSD which was not statistically significant. This is likely because the ONSD is a surrogate for intracranial pressure, whereas the ICH score is a mortality marker. Not all patients with a high intracranial pressure have a poor outcome and vice versa. 

There are several limitations to this study. During enrolment of patients, the CT head was viewed prior to measuring the optic nerves. This potentially created a bias as the physician looking at the imaging and performing the ultrasound exam was the same individual. 27 of 28 ultrasound exams were performed by the same physician, and one exam was performed by another physician; therefore, inter-rater reliability was not assessed, which potentially limits the generalization of the conclusion. 

## Conclusion

Measuring the optic nerve sheath diameter with POCUS is a quick noninvasive tool that can be used in the context of acute intracerebral hemorrhage to assess the volume of an ICH at a given point in time. We found a moderate positive correlation between the ONSD and volume of hematoma. This may be taken further, to monitor the stability or expansion of a hematoma indirectly, although this is a potential application of this technique and was not directly examined in this study. This POCUS exam is particularly useful in populations where performing neurological exams is challenging due to sedation or lack of patient cooperation. The ONSD is not meant to replace the physical exam but rather be an objective adjunct tool. A future study needs to be performed where serial measurements of ONSD are performed and compared with ICH volume. 

## Disclosures

The authors report no disclosures related to this work.
